# Long-Term Results with 187 Frozen Elephant Trunk Procedures

**DOI:** 10.3390/jcm12124143

**Published:** 2023-06-20

**Authors:** Zsuzsanna Arnold, Daniela Geisler, Thomas Aschacher, Bernhard Winkler, Verena Lenz, Ingo Crailsheim, Sandra Folkmann, Marieluise Harrer, Reinhard Moidl, Martin Grabenwöger, Gabriel Weiss

**Affiliations:** 1 Department of Cardiovascular Surgery, Clinic Floridsdorf, 1210 Vienna, Austria; 2 Institute of Cardiovascular Research, Karl Landsteiner Society, 1210 Vienna, Austria; 3 Medical Faculty, Sigmund Freud University, 1020 Vienna, Austria

**Keywords:** frozen elephant trunk, aortic arch, aortic dissection, aortic aneurysm

## Abstract

The frozen elephant trunk (FET) technique is an established therapeutic option in the treatment of complex aortic diseases. We report our long-term clinical outcomes after FET repair. A total of 187 consecutive patients underwent FET repair at our department between 8/2005 and 3/2023. Indications included acute and chronic aortic dissections and thoracic aneurysms. Endpoints included operative morbidity and mortality, long-term survival, and the need for reinterventions. Operative mortality, spinal cord injury and permanent stroke rates were: 9.6%, 2.7% and 10.2%, respectively. At five years, overall survival was 69.9 ± 3.9% and freedom from aortic-related death was 82.5 ± 3.0%, whereas at ten years, overall survival was 53.0 ± 5.5% and freedom from aortic-related death was 75.8 ± 4.8%. Sixty-one reinterventions on the thoracic aorta were necessary. Freedom from secondary interventions at ten years was 44.7 ± 6.4% overall (63.1 ± 10.0% for acute dissections, 40.8 ± 10.3% for chronic dissections and 28.9 ± 13.1% for aneurysms, respectively). The high reintervention rate for chronic dissections and for aneurysms is related to the pre-existing aortic pathology. Late aortic growth of untreated segments with potentially fatal outcome occurs even after ten years, so careful annual follow-up is mandatory in this patient cohort.

## 1. Introduction

The frozen elephant trunk (FET) procedure has become the primary therapeutic option for complex aortic pathologies involving the arch and the proximal descending aorta [1]. As the FET technique has gained widespread application, numerous surgical strategies have been described, with various clinical outcomes [2,3,4,5,6]. The long-term data in an unselected, consecutive patient cohort support the decision-making process in elective as well as in emergent cases of aortic syndromes. At our center, we exclusively use the E-vita Open (E-vita Open, E-vita Open Plus and E-vita Open NEO) Stent Graft System (Jotec, Hechingen, Germany). Indications for the FET procedure include acute and chronic type A and B dissections of the aorta as well as aortic aneurysms with involvement of the arch and/or proximal descending aorta.

As the distal end of the stent graft offers an optimal landing zone for secondary interventions, FET repair is often performed as a planned first-stage procedure in the treatment of thoracoabdominal aneurysms [7,8,9]. In the case of Stanford Type A aortic dissections, the FET technique is applied when re-entries are detected in the distal aortic arch or proximal descending aorta. A descending aorta diameter exceeding 40 mm, with which the possible need for later treatment increases, also represents an indication for the FET procedure. Type B and non-A–non-B dissections with indication for therapy are treated with a FET prosthesis if the arch is not suitable for endovascular repair.

The aim of this study was to report our long-term clinical outcomes after FET repair.

## 2. Materials and Methods

### 2.1. Patient Population

This retrospective study was approved by the local ethics committee (approval number: EK 20-326-VK).

Between August 2005 and March 2023, 187 consecutive patients underwent FET repair at our department. At the beginning, the E-vita Open, since 2008 the E-vita Open Plus and from 2020 on the E-vita Open NEO hybrid graft (Jotec, Hechingen, Germany) have been used in this cohort of patients. Indications included thoracic aortic aneurysms (n = 74; 39.6%), acute aortic dissections (n = 64; 34.2%) and chronic aortic dissections (n = 49; 26.2%) with involvement of the aortic arch and/or the proximal descending aorta.

Preoperative patient characteristics are demonstrated in Table 1.

In-hospital data were collected retrospectively. Outpatient follow-up with a computed tomography (CT) scan was carried out routinely as recommended in the respective guidelines [10]. Clinical data were analyzed according to the underlying pathology (aneurysms, acute (AAD) and chronic aortic dissections (CAD)).

Aortic dissection was defined as acute if surgery was performed in less than 14 days after the onset of pain and CT diagnosis [11].

### 2.2. Operative Strategy

Our surgical technique has been previously described elsewhere [12,13]. Over the study period, perioperative management was adapted according to emerging experience.

Since 2014, we have performed the anastomosis of the FET prosthesis cuff predominantly in zone 2, as we experienced several advantages with this method. Besides better exposure and improved hemostasis, the risk of left laryngeal nerve injury decreases as well. In cases of unfavorable anatomical conditions, we performed left subclavian artery (LSA) revascularization to facilitate deployment of the hybrid prosthesis in zone 2. The LSA was ligated at its offspring of the arch, thus avoiding type II endoleak formation. LSA revascularization was completed either electively as a previous left carotid-subclavian bypass (n = 48), or in urgent cases concomitantly as an extraanatomical aorto-subclavian bypass (n = 21).

The level of proximal stent-graft anastomosis was zone 1 in four cases, zone 2 in 76 cases, zone 3 in 105 cases and zone 4 in two cases. Total arch replacement was performed in 102 patients (54.5%), whereas 80 patients (42.8%) received a partial arch repair, previously described as the “arch-light” technique [12,13]. In cases of no large arch aneurysms (maximal diameter <50 mm) or connective tissue disorders, the arch-light technique was favored. In the absence of an indication for arch replacement, the FET stent-graft was inserted via direct incision of the arch (n = 5; 2.7%).

Since May 2018, we have adopted the technique of descending perfusion for total arch repair. Initially, we cannulated the descending aorta directly with an aortic balloon occlusion catheter with perfusion lumen, until the E-vita Open NEO grafts with a side branch for distal perfusion became available. Moderate hypothermic cardiac arrest (MHCA) and selective antegrade bilateral cerebral perfusion (SACP) were carried out as the institutional standard. Upon completing the distal anastomosis in zone 1–4, we clamp the prosthesis in the height of the ascending aorta. All side branches are clamped as well. An arterial canula is inserted into the distal perfusion side branch and descending perfusion is commenced. We maintain the patient in a moderately hypothermic state throughout the duration of selective anterior cerebral perfusion for the implantation of the supraaortic vessels. We routinely start restoring normal flow on cardiopulmonary bypass and warming up patients upon completion of cerebral vessel reimplantation.

Cerebrospinal fluid drainage (CSF) was applied in selected cases of high risk for spinal cord ischemia (following long stretched thoracic endovascular aortic repair (TEVAR) or open thoraco-abdominal aortic aneurysm (TAAA) repair; n = 2) and postoperatively in one case of acute type B dissection.

To minimize the risk of peripheral vascular complications, we abandoned the concept of retrograde guide wire insertion and placed the guide wire solely antegrade through the opened aortic arch under angioscopy control.

Intraoperative details are given in Table 2.

### 2.3. Endpoints

Endpoints included operative morbidity and mortality, long-term overall and aortic-related survival as well as the incidence of secondary interventions. Any death occurring within 30 days after the index procedure was considered to be operative mortality. Any death occurring before hospital discharge was considered in-hospital mortality. Aortic-related death was defined as any mortality caused by an aortic pathology or as a consequence of the primary or a secondary intervention. Stroke was defined as permanent focal neurological deficit and/or confirmation of focal cerebral lesion by imaging (CT), including patients with cerebral malperfusion. Spinal cord injury (SCI) was defined as permanent paraparesis or paraplegia, including cases of spinal malperfusion in acute dissections [11]. Secondary interventions included all elective and emergent, open and endovascular procedures performed on the thoracic aorta. The indication for elective reintervention was given if the maximal diameter of the aorta exceeded 55 mm or the annual expansion exceeded 3 mm. Emergent reinterventions were performed in case of acute aortic rupture, prosthesis infection or re-dissection. Endovascular treatment was preferred in all feasible cases except for patients with connective tissue disease [14].

### 2.4. Statistical Analysis

Categorical variables are reported as total numbers (n) and frequency (percentage), whereas continuous variables are reported as mean ± standard deviations (range) or as median (interquartile range). Survival and event-free survival curves were estimated by the Kaplan–Meier method; the date of the index procedure was designated as time zero. Categorical data were compared using Pearson’s chi-squared test. Normally distributed continuous variables were compared using the analysis of variance test (ANOVA) and the Tukey–Kramer pairwise post hoc test; in the case of a non-normal distribution, either the Mann–Whitney U test or the Kruskal–Wallis rank test with the Shaffer correction for pairwise comparisons was applied. The tests were two-sided, and *p*-values < 0.05 were considered to be significant. IBM SPSS Statistics 27 (IBM, Armonk, NY, USA) and GraphPad Prism 9 (GraphPad Software, San Diego, CA, USA) software was used for statistical analysis [15].

## 3. Results

### 3.1. Early Outcomes

The intraoperative data shown in Table 2 demonstrate that, despite more concomitant procedures, aneurysm surgeries were associated with shorter ischemia, cardiopulmonary bypass, and skin-to-skin times than dissections. The chosen operative technique significantly influenced ischemia times: total arch replacement was associated with longer mean myocardial ischemia time and SACP time than partial or no arch replacement (cross-clamp time for total and partial or no arch repair: 113 ± 32 min vs. 101 ± 28 min, respectively, *p* = 0.011; SACP time: 65 ± 18 min, vs. 51 ± 12 min, *p* < 0.001). On the other hand, visceral ischemia time did not differ significantly in these groups, which is explained by the frequent use of descending perfusion for total arch repair (51 ± 20 min vs. 55 ± 13 min, *p* = 0.207). In the era previously to the implementation of distal perfusion, total arch repair was associated with significantly longer visceral ischemia time than after introducing descending perfusion routinely (mean visceral ischemia time: 64 ± 21 min vs. 40 ± 9 min; *p* < 0.001); whereas mean SACP time increased slightly: 60 ± 20 min vs. 69 ± 13 min; *p* = 0.032).

Detailed data on clinical outcomes are given in Table 3.

The 30-day mortality rate was 9.1% (n = 17), whereas the in-hospital mortality rate was 12.8% (n = 24). Causes of death included multiorgan failure (n = 11), cerebral herniation due to stroke (n = 6), pneumonia (n = 2), cardiogenic shock (n = 2), uncontrollable bleeding due to severe coagulopathy (n = 2) and re-dissection with cardiac tamponade (n = 1). Thirteen of these patients presented with an aortic dissection (nine with AAD and four with CAD). Both patients who died due to cardiac failure had a history of cardiomyopathy with a preoperative left ventricular ejection fraction below 20%.

The perioperative permanent stroke rate was 10.2% (n = 19). Acute dissections were associated with the highest stroke rate (n = 9; 14.1%), followed by chronic dissections (n = 4; 8.2%) and aneurysms (n = 6; 8.1%; *p* = 0.464). Out of six patients with fatal stroke, one presented with cerebral malperfusion, and the other five patients were neurologically asymptomatic at the time of admission.

The SCI rate was 2.7% (n = 5). Two of these patients died: one on the first day after surgery, and the other patient on postoperative day 107. Two other patients had transient symptoms. One patient, who was initially admitted with paraplegia as a result of spinal malperfusion in AAD, was discharged with permanent paraparesis. The only patient who developed this complication despite perioperative CSF drainage had an extensive TAAA (type I by Crawford classification) and was previously treated with multiple stent-grafts in a TEVAR procedure. Mean visceral ischemia times did not differ significantly in patients with or without SCI (45.8 ± 4.1 min vs. 53.1 ± 17.3 min, *p* = 0.350).

Follow-up was 98.9% complete (n = 185 out of 187 patients), at a mean of 4.2 ± 4.1 years (range 0.01–17.5 years) with 787.4 patient years. Follow-up CT angiograms were available for 159 out of 163 discharged patients (97.5%).

### 3.2. Late Mortality

**Figure 1:**
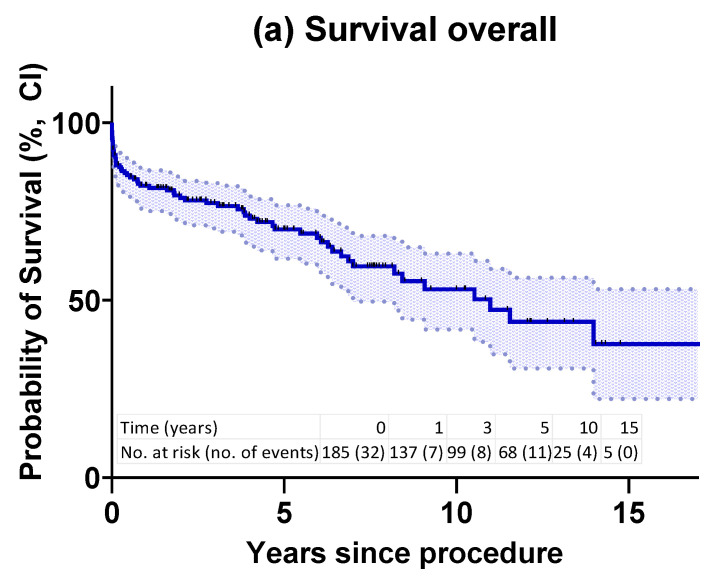

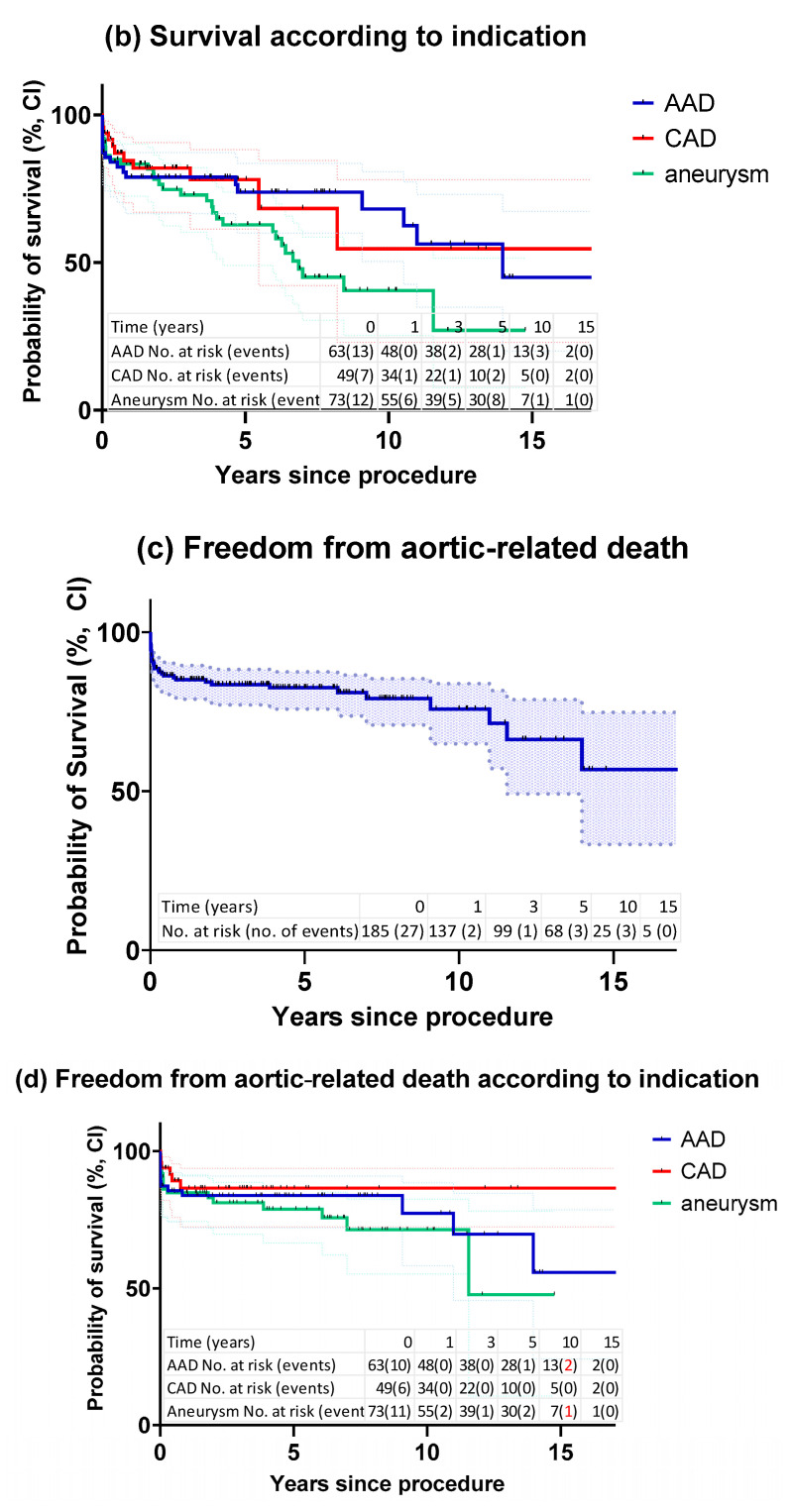
(**a**): Kaplan–Meier overall survival estimate in all patients. (**b**): Kaplan–Meier survival estimate according to indication for FET procedure. Log-rank (Mantel–Cox) test *p* = 0.1299. (**c**): Kaplan–Meier estimate of freedom from aortic-related death in all patients. (**d**): Kaplan–Meier estimate of freedom from aortic-related death according to indication for FET procedure. Log-rank (Mantel–Cox) test *p* = 0.4167. AAD: acute aortic dissections (blue); CAD: chronic aortic dissections (red); aneurysms (green). CI: confidence interval; FET: frozen elephant trunk.

Kaplan–Meier overall survival rates at one, five, ten and 15 years were 82%, 70%, 53% and 38%, respectively. Freedom for aortic-related death was 85%, 83%, 76% and 57% at one, five, ten and 15 years, respectively (Figure 1a–d).

Red numbers in Figure 1d represent three cases of very late aortic mortality. Causes of late aortic mortality included aortic rupture (n = 3), aorto-esophageal fistula (n = 1), aorto-bronchial fistula (n = 1), prosthesis infection (n = 2), prosthesis material rupture (n = 1) and complications following open thoracoabdominal aortic repair (n = 2).

### 3.3. Secondary Aortic Interventions

**Figure 2:**
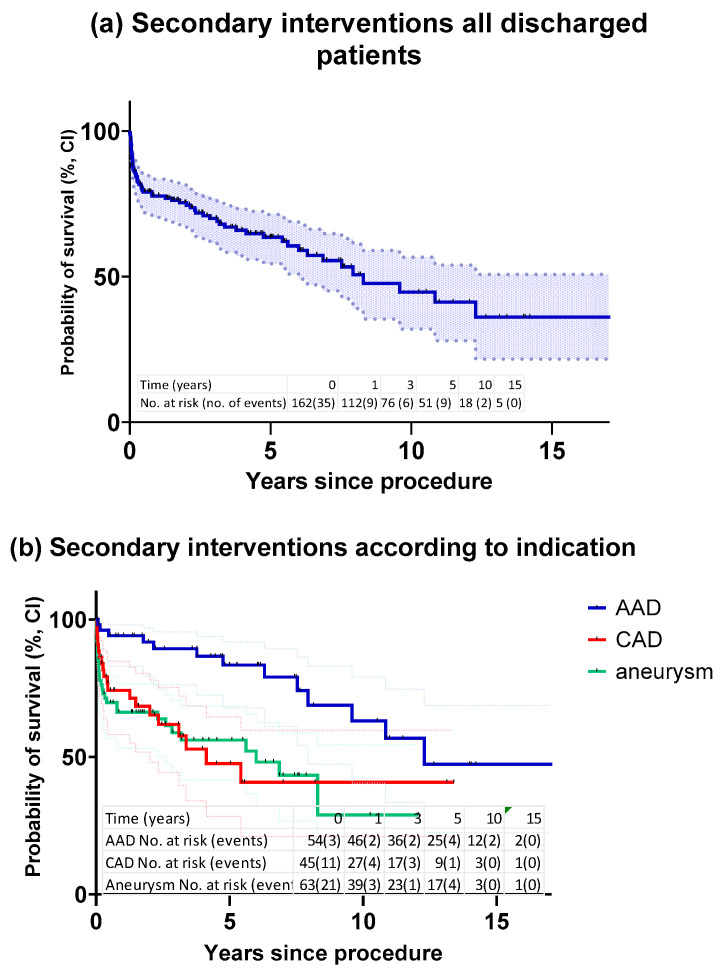
(**a**): Kaplan–Meier estimate of freedom from secondary interventions in all discharged patients in follow-up (n = 162). (**b**): Kaplan–Meier estimate of freedom from secondary interventions according to indication for FET procedure. AAD: acute aortic dissections (blue); CAD: chronic aortic dissections (red); aneurysms (green). Log-rank (Mantel–Cox) test *p* = 0.0012. CI: confidence interval; FET: frozen elephant trunk.

A total of 61 patients required reintervention of the thoracic aorta. Freedom from secondary interventions at ten years was highest after acute dissection (63.1%), followed by chronic dissection (40.8%) and aneurysm (28.9%; Figure 2a,b). Correspondingly, the mean time to the first reintervention according to the initial FET indication was longest in the acute dissection cohort, followed by the aneurysms and the chronic dissections (5.2, 1.4 and 1.3 years, respectively, and 2.2 years overall; *p* < 0.001).

Five urgent or emergent reinterventions included one TEVAR, one open TAAA repair and one hybrid arch debranching for acute rupture of the descending aorta, one Bentall-procedure due to re-dissection of the aortic root and one replacement of the FET prosthesis with bioprosthetic due to graft infection. One of these patients, whose aortic rupture occurred 11 years after the index procedure, died of multiorgan failure after the emergent hybrid reintervention. The other four patients were discharged from the hospital after an uneventful postoperative course.

The 56 elective secondary interventions were planned and indicated by a large or growing diameter of the distal arch or descending aorta. Endovascular treatment could be performed in 43 patients (76.8%). Large TAAAs (type I by Crawford classification) were treated with open TAAA repair in 11 cases (19.6%). Two patients needed redo sternotomy due to expansion of the distal arch: one of them underwent a redo FET repair, whereas the other received hybrid arch debranching.

Three of the four redo arch repairs (two hybrid debranchings and one redo FET) were necessary in patients who previously underwent only partial or no arch replacement. One patient needed complete replacement of all alloplastic material via a biological prosthesis due to severe infection and mediastinitis.

Procedural serious adverse events (including death and new onset stroke or SCI) rates following elective endovascular and open secondary interventions did not differ (n = 5 out of 43 TEVARs; 11.6% vs. n = 2 out of 13 open surgeries; 15.4%; *p* = 0.7197).

Permanent hemodialysis was required for three patients following open TAAA repair (25%) and for none after TEVAR.

## 4. Discussion

The 30-day mortality rate of 9.1% is comparable to previously reported early mortality rates following FET repair (4–27%) [3,5,16,17,18,19,20,21]. Compared with the other published series, the incidence of permanent SCI in our study cohort was low, with a rate of 2.7%. This low incidence may be associated with the use of the shorter E-vita Open hybrid graft with a maximum stent graft length of 12 or 13 cm in the majority of the cases and the use of CSF drainage in selected patients. However, previous studies failed to establish a link between the length of the FET prosthesis and perioperative outcome [3].

Due to evolving surgical techniques and perioperative management, periprocedural morbidity and mortality have been improving steadily over the years, so that attention is now shifting towards late outcomes, especially towards the monitoring and treatment of late aortic growths and endoleak formation. The FET stent graft can be deployed either as part of a total arch replacement or with the previously described “arch-light” technique (or peninsula style) [3,12]. For the latter, upon deploying the stented graft, the vascular prosthesis is pulled out of it and shortened to a rim of approximately 2 cm, which is then sutured to the aortic wall in zone 3. The preserved connection between the supraaortic branches and the descending aorta enables a straightforward partial arch repair with a second vascular prosthesis. We observed significantly shorter myocardial ischemia and SACP times with this simplified technique than with traditional total arch repair.

Procedures for acute and chronic aortic dissections were associated with significantly longer ischemic and operative times than aneurysm surgeries. FET implantation in a dissected aorta is even more challenging than in aneurysms due to the vulnerability of the tissue at the site of the anastomosis and the often-strenuous identification of true and false lumina. Concomitant procedures involving the aortic root were repeatedly necessary in AAD and CAD cases, whereas aneurysm patients were more frequently treated with CABG. The complexity of root surgery could have also contributed to the longer surgeries in dissection patients.

However, following FET repair, complications proximal to the stent graft are rare and, in our experience, associated exclusively with untreated aortic segments. Thus, the extent of arch replacement (total vs. partial) must be considered carefully on an individual basis when aiming for the optimal surgical strategy. Three out of four patients who received redo surgeries involving the aortic arch underwent previously partial arch replacement and the only case of acute re-dissection of the aortic root occurred after supracoronary replacement of the ascending aorta. Upon these events, we modified our surgical strategy by further restricting partial arch replacement in cases where the distal arch (distal of zone 2) and especially the convexity of the arch was not affected by any aortic pathology (specifically no connective tissue disorder, maximal diameter < 35 mm, no intimal lesion and lack of calcifications). The technique of distal perfusion further facilitated the performance of total arch repair and shortened spinal and lower body hypothermic circulatory arrest times.

Recently, we modified our surgical technique by moving the proximal FET stent-graft landing zone from zone 3 to 2. Besides technically facilitating the proximal suture line, this has several advantages. The often-challenging reimplantation of the LSA can be performed whilst perfusing the descending aorta, which shortens the circulatory arrest time. To further reduce the risk of laryngeal palsy, we favor the extraanatomical implantation of a Dacron prosthesis between any part of the central portion of the E-vita prosthesis and the infraclavicular LSA. In elective cases, such as chronic dissections and aneurysms, we prefer to perform a bypass from the left common carotid artery to the LSA first. The previous revascularization of the LSA facilitates a total arch replacement and, therefore, minimizes the risk of developing a type II endoleak.

In our series, secondary interventions were associated with considerable periprocedural mortality and morbidity (19.6% for both endovascular and open reinterventions vs. 14–40% reported in the literature [9,22,23]). Our results continue to fuel the as-yet-unsolved debate on the extent of surgical invasiveness. Increasing operative complexity is associated with worse short-term morbidity and mortality, whereas untreated segments of a diseased aorta are prone to dissection, rupture, and late growth. Treating as much of the aorta as possible in the acute phase of the disease facilitates complete remodeling by closure of re-entries and excluding the false lumen in the proximal descending aorta [24]. Chronic dissections present with a thickened dissection membrane. In these cases, the purpose of FET treatment is to establish a proper landing zone for further endovascular or open surgical therapies. Although avoiding extensive interventions for better short-term results may be reasonable for the critical patient or in the urgent setting, in elective cases—such as chronic dissections or aneurysms—a multidisciplinary, individually tailored strategy with the treatment of as much pathology as possible is needed to prevent emergent redo operations or high-risk endovascular procedures. Therefore, the novel FET prosthesis with a stented side branch for the LSA could provide a simplification of total arch replacement while maintaining the long-term advantages of treating all affected aortic arch segments [25].

Consequently, we treated all eleven patients with connective tissue disorders with complete arch replacement. Eight of these patients already had a history of root replacement, whereas in three cases we performed a Tirone David (n = 1) or a modified Bentall–De Bono procedure (n = 2) for the treatment of the aortic root at the same time as the FET repair. None of them needed emergent reintervention during follow-up, and two planned open TAAA repairs were uneventful. One patient with Loeys–Dietz syndrome needed repeated reinterventions over the years due to recurrent endoleaks despite uncomplicated surgical treatment of the entire aorta. These results provide further evidence that the FET stent graft offers an optimal landing zone for secondary interventions even in genetic aortic syndromes, especially because the FET endoprosthesis is surgically fixed and, therefore, unlike a normal TEVAR, can be used relatively safely in connective tissue disorders. The use of endovascular or hybrid techniques to avoid or bridge to (repeat) surgery is now supported by emerging data in this patient cohort as well [26,27,28].

Although initially developed as a potential single-stage procedure [6], the FET technique has gained increasing attention as part of multi-stage treatment strategies [8]. Similar to published experiences, 76.5% of our elective secondary interventions could be performed endovascularly [29]. Since Etz et al. reported a significant reduction in SCI with two-stage vs. single-stage thoracoabdominal aneurysm repair, many centers have applied the FET prosthesis as the initial stage of complex TAAA treatments [30]. In our department, to allow the collateral network to develop, secondary interventions on the downstream aorta are performed as staged procedures within a minimum interval of 8–12 weeks after the FET repair, whenever possible.

Many studies have reported higher reintervention rates in patients with aneurysms or chronic dissections compared with acute dissection cases [4,17,29,31]. In fact, most of the secondary interventions in our patient cohort were planned as a second-stage repair at the time of the index procedure.

Notably, aortic-related mortality occurs even after years of stability in aortic diameters distal or proximal to already-treated segments. Untreated aortic segments could start to grow and eventually rupture even after long uneventful periods (in our series ranging between 1.8 and 10.8 years), underlying the need for lifelong annual follow-up controls of patients with aortic syndromes.

## 5. Conclusions

The frozen elephant trunk technique has become the primary therapeutic option in extensive thoracic aortic diseases involving the arch and/or the proximal descending aorta. The high reintervention rate for chronic dissections as well as for thoracic aneurysms is mostly related to pre-existing aortic pathologies. Late growth of the remaining untreated aorta with possible fatal outcome occurs even after ten years; therefore, careful follow-up and timely planning of reinterventions are mandatory in this patient cohort.

## 6. Limitations

The obvious limitations of the study are, besides its retrospective observational character, the relatively small number of patients, especially in the chronic dissection subgroup, and the single center design. During the long study period, with growing evidence and experience, we modified our surgical strategy and perioperative management, which could have influenced the results. However, further analysis according to different techniques and treatments would have resulted in smaller subgroup sizes with possibly poor-quality data.

## Figures and Tables

**Table 1:** Demographics and preoperative data.

	All	AAD	CAD	Aneurysm	*p*-Value
	n = 187 (100%)	n = 64 (34.2%)	n = 49 (26.2%)	n = 74 (39.6%)	
Age (years), mean ± SD	62.1 ± 12.1	58.9 ± 11.8	56.9 ± 12.9	68.3 ± 8.9	<0.001
Male	104(55.6)	49 (76.6)	26 (53.1)	29 (39.2)	<0.001
Diagnosis					
Aneurysm	74 (39.6)	0	0	74 (100)	NC
Acute aortic dissection	64 (34.2)	64 (100)	0	0	NC
Type A	45 (24.1)	45 (70.3)	0	0	NC
Non-A–non-B	8 (4.3)	8 (12.5)	0	0	NC
Type B	11 (5.9)	11 (17.2)	0	0	NC
Chronic aortic dissection	49 (26.2)	0	49 (100)	0	NC
Type A	20 (10.7)	0	20 (40.8)	0	NC
Non-A–non-B	13 (7.0)	0	13 (26.5)	0	NC
Type B	16 (8.6)	0	16 (32.7)	0	NC
Marfan syndrome	8 (4.3)	3 (4.7)	5 (10.2)	0	0.016
Loeys–Dietz syndrome	2 (1.1)	0	1 (2.0)	1 (1.4)	0.728
Turner syndrome	1 (0.5)	1 (1.6)	0	0	0.604
Redo operation	31 (16.6)	2 (3.1)	26 (53.1)	3 (4.1)	<0.001
Previous left carotid-subclavian bypass	48 (25.7)	9 (14.1)	23 (46.9)	16 (21.6)	<0.001
Previous intervention of descending aorta	32 (17.1)	8 (12.5)	10 (20.4)	14 (17.1)	0.471
Endovascular	23 (12.3)	8 (12.5)	5 (10.2)	10 (13.5)	0.888
Open	9 (4.8)	0	5 (10.2)	4 (5.4)	0.033
Malperfusion in 113 (100%) aortic dissections	17 (15.0)	14 (21.9)	3 (6.1)	0	0.060
Cerebral and spinal	4 (3.5)	4 (6.3)	0	0	NC
Spinal	1 (0.9)	1 (1.6)	0	0	NC
Mesenteric	5 (4.4)	5 (7.8)	0	0	NC
Renal	1 (0.9)	1 (1.6)	0	0	NC
Lower limb	6 (5.3)	3 (4.7)	3 (6.1)	0	1.000

AAD: acute aortic dissection; CAD: chronic aortic dissection; SD: standard deviation.

**Table 2:** Intraoperative data.

	All	AAD	CAD	Aneurysm	*p*-Value
	n = 187 (100%)	n = 64 (34.2%)	n = 49 (26.2%)	n = 74 (39.6%)	
Operation time (min), mean ± SD	347 ± 66	370 ± 81	357 ± 59	323 ± 45	<0.001
Cardiopulmonary bypass time (min), mean ± SD	200 ± 44	212 ± 55	212 ± 44	183 ± 26	<0.001
SACP time (min), mean ± SD	58 ± 17	62 ± 19	62 ± 15	52 ± 14	<0.001
Visceral ischemia time (min), mean ± SD	53 ± 17	62 ± 21	50 ± 14	47 ± 11	<0.001
Cardiac ischemia time (min), mean ± SD	108 ± 31	113 ± 33	112 ± 33	100 ± 25	0.019
Descending perfusion (min), mean ± SD *	29 ± 9	28 ± 8	31 ± 10	28 ± 9	0.622
Proximal stent-graft landing zone					
Zone 1	4 (2.1)	0 (0)	3 (6.1)	1 (1.4)	0.625
Zone 2	76 (40.6)	18 (28.1)	31 (63.3)	27 (36.5)	0.174
Zone 3	105 (56.1)	45 (70.3)	15 (30.6)	45 (60.8)	<0.001
Zone 4	2 (1.1)	1 (1.6)	0 (0)	1 (1.4)	1
Arch replacement					
Total	102 (54.5)	25 (39.1)	41 (83.7)	36 (48.6)	0.139
Partial	80 (42.8)	38 (59.4)	6 (12.2)	36 (48.6)	<0.001
None	5 (2.7)	1 (1.6)	2 (4.1)	2 (2.7)	1
Ascending aorta and aortic root interventions					
Modified Bentall–de Bono procedure	9 (4.8)	5 (7.8)	2 (4.1)	2 (2.7)	
Tirone David procedure	12 (6.4)	3 (4.7)	4 (8.2)	5 (6.8)	
AVR with supracoronary ascending replacement	14 (7.5)	3 (4.7)	3 (6.1)	8 (10.8)	
Ascending replacement	146(78.1)	52 (81.3)	37 (75.5)	57 (77.0)	
None	6 (3.2)	1 (1.6)	3 (6.1)	2 (2.7)	
Concomitant procedures					
CABG	27 (14.4)	0 (0)	3 (6.1)	24 (32.4)	
Mitral or tricuspid repair	4 (2.1)	0 (0)	2 (4.1)	2 (2.7)	
Maze procedure	1 (0.5)	0 (0)	0 (0)	1 (1.4)	
Intraoperative TEVAR	6 (3.2)	5 (7.8)	0 (0)	1 (1.4)	

AAD: acute aortic dissection; AVR: aortic valve replacement; CABG: coronary artery bypass grafting; CAD: chronic aortic dissection; SACP: selective antegrade cerebral perfusion, SD: standard deviaton; TEVAR: thoracic endovascular aortic repair; * 52 cases of descending perfusion.

**Table 3:** Postoperative outcome.

	All	AAD	CAD	Aneurysm	*p*-Value
	n = 187 (100%)	n = 64 (34.2%)	n = 49 (26.2%)	n = 74 (39.6%)	
30 days mortality	17 (9.1)	8 (12.5)	3 (6.1)	6 (8.1)	0.474
In-hospital mortality	24 (12.8)	9 (14.1)	4 (8.2)	11 (14.9)	0.533
Prolonged ventilation (>72 h)	49 (26.2)	22 (34.4)	13 (26.5)	14 (18.9)	0.120
Stroke	19 (10.2)	9 (14.1)	4 (8.2)	6 (8.1)	0.464
Paraparesis	5 (2.7)	1 (1.6)	2 (4.1)	2 (2.7)	0.852
Dialysis					
permanent	4 (2.1)	1 (1.6)	1 (2.0)	2 (2.7)	1
temporary	30 (16.0)	14 (21.9)	8 (16.3)	8 (10.8)	0.210
Recurrent nerve palsy	8 (4.4)	1 (1.6)	4 (8.2)	3 (4.1)	0.280
Rethoracotomy for bleeding or infection	20 (10.7)	8 (12.5)	5 (10.2)	7 (9.5)	0.840
ICU stay (days), mean ± SD	10.3 ± 18.0	11.4 ± 13.5	10.4 ± 14.5	9.4 ± 22.9	0.825
Hospital stay (days) mean ± SD	29 ± 30	36 ± 37	26 ± 21	26 ± 30	0.127
Patients in follow-up	186 (99.5)	63	49	74	
Follow-up time (years), mean ± SD	4.2 ± 4.1	5.1 ± 4.6	3.3 ± 3.8	4.1 ± 3.6	0.066
Secondary interventions	62 (33.2)	14 (21.9)	19 (38.8)	29 (39.2)	0.061
Open TAAA repair	12 (6.4)	0 (0)	4 (8.2)	8 (10.8)	0.030
TEVAR	45 (24.1)	10 (15.6)	15 (30.6)	20 (27.0)	0.135
Aortic root/arch	5 (2.7)	4 (6.3)	0 (0)	1 (1.4)	0.074
Time to first re-intervention (years), mean ± SD	2.2 ± 3.1	4.8 ± 4.3	1.3 ± 1.6	1.4 ± 2.4	<0.001

AAD: acute aortic dissection; CAD: chronic aortic dissection; ICU: intensive care unit; SD: standard deviaton; TAAA: thoraco-abdominal aortic aneurysm; TEVAR: thoracic endovascular aortic repair.

## Data Availability

Data are available upon request.

## References

[1] Czerny M., Schmidli J., Adler S., van den Berg J.C., Bertoglio L., Carrel T., Chiesa R., Clough R.E., Eberle B., Etz C. (2019). Current options and recommendations for the treatment of thoracic aortic pathologies involving the aortic arch: An expert consensus document of the European Association for Cardio-Thoracic surgery (EACTS) and the European Society for Vascular Surgery (ESVS). Eur. J. Cardiothorac. Surg..

[2] Czerny M., Rylski B., Kari F.A., Kreibich M., Morlock J., Scheumann J., Kondov S., Südkamp M., Siepe M., Beyersdorf F. (2017). Technical details making aortic arch replacement a safe procedure using the Thoraflex Hybrid prosthesis. Eur. J. Cardiothorac. Surg..

[3] Berger T., Weiss G., Voetsch A., Arnold Z., Kreibich M., Rylski B., Krombholz-Reindl P., Winkler A., Mach M., Geisler D. (2019). Multicentre experience with two frozen elephant trunk prostheses in the treatment of acute aortic dissectiondagger. Eur. J. Cardiothorac. Surg..

[4] Leone A., Beckmann E., Martens A., Di Marco L., Pantaleo A., Reggiani L.B., Haverich A., Di Bartolomeo R., Pacini D., Sherestha M. (2020). Total aortic arch replacement with frozen elephant trunk technique: Results from two European institutes. J. Thorac. Cardiovasc. Surg..

[5] Di Bartolomeo R., Di Marco L., Armaro A., Marsilli D., Leone A., Pilato E., Pacini D. (2009). Treatment of complex disease of the thoracic aorta: The frozen elephant trunk technique with the E-vita open prosthesis. Eur. J. Cardiothorac. Surg..

[6] Karck M., Chavan A., Hagl C., Friedrich H., Galanski M., Haverich A. (2003). The frozen elephant trunk technique: A new treatment for thoracic aortic aneurysms. J. Thorac. Cardiovasc. Surg..

[7] Folkmann S., Weiss G., Pisarik H., Czerny M., Grabenwoger M. (2015). Thoracoabdominal aortic aneurysm repair after frozen elephant trunk procedure. Eur. J. Cardiothorac. Surg..

[8] Gkremoutis A., Zierer A., Schmitz-Rixen T., El-Sayed Ahmad A., Kaiser E., Keese M., Schmandra T. (2017). Staged treatment of mega aortic syndrome using the frozen elephant trunk and hybrid thoracoabdominal repair. J. Thorac. Cardiovasc. Surg..

[9] Haensig M., Schmidt A., Staab H., Steiner S., Scheinert D., Branzan D. (2020). Endovascular repair of the thoracic or thoracoabdominal aorta following the frozen elephant trunk procedure. Ann. Thorac. Surg..

[10] Erbel R., Aboyans V., Boileau C., Bossone E., Di Bartolomeo R., Eggebrecht H., Evangelista A., Falk V., Frank H., Gaemperli O. (2014). 2014 ESC guidelines on the diagnosis and treatment of aortic diseases. Kardiol. Pol. (Pol. Heart J.).

[11] Rylski B., Pacini D., Beyersdorf F., Quintana E., Schachner T., Tsagakis K., Ronchey S., Durko A., De Paulis R., Siepe M. (2019). Standards of reporting in open and endovascular aortic surgery (STORAGE guidelines). Eur. J. Cardiothorac. Surg..

[12] Gorlitzer M., Weiss G., Thalmann M., Mertikian G., Wislocki W., Meinhart J., Waldenberger F., Grabenwogerm M. (2007). Combined surgical and endovascular repair of complex aortic pathologies with a new hybrid prosthesis. Ann. Thorac. Surg..

[13] Weiss G., Santer D., Dumfarth J., Pisarik H., Harrer M.L., Folkmann S., Mach M., Moidl R., Grabenwoger M. (2016). Evaluation of the downstream aorta after frozen elephant trunk repair for aortic dissections in terms of diameter and false lumen status. Eur. J. Cardiothorac. Surg..

[14] Grabenwöger M., Alfonso F., Bachet J., Bonser R., Czerny M., Eggebrecht H., Evangelista A., Fattori R., Jakob H., Lönn L. (2012). Thoracic Endovascular Aortic Repair (TEVAR) for the treatment of aortic diseases: A position statement from the European Association for Cardio-Thoracic Surgery (EACTS) and the European Society of Cardiology (ESC), in collaboration with the European Association of Percutaneous Cardiovascular Interventions (EAPCI). Eur. Heart J..

[15] Hickey G.L., Dunning J., Seifert B., Sodeck G., Carr M.J., Burger H.U., Beyersdorf F. (2015). Statistical and data reporting guidelines for the European Journal of Cardio-Thoracic Surgery and the Interactive CardioVascular and Thoracic Surgery. Eur. J. Cardiothorac. Surg..

[16] Shrestha M., Kaufeld T., Beckmann E., Fleissner F., Umminger J., Abd Alhadi F., Boethig D., Krueger H., Haverich A., Martens A. (2016). Total aortic arch replacement with a novel 4-branched frozen elephant trunk prosthesis: Single-center results of the first 100 patients. J. Thorac. Cardiovasc. Surg..

[17] Jakob H., Dohle D., Benedik J., Janosi R.A., Schlosser T., Wendt D., Thielmann M., Erbel R., Tsagakis K. (2017). Long-term experience with the E-vita Open hybrid graft in complex thoracic aortic disease. Eur. J. Cardiothorac. Surg..

[18] Di Eusanio M., Pantaleo A., Murana G., Pellicciari G., Castrovinci S., Berretta P., Folesani G., Di Bartolomeo R. (2013). Frozen elephant trunk surgery-the Bologna’s experience. Ann. Cardiothorac. Surg..

[19] Shi E., Gu T., Yu Y., Yu L., Wang C., Fang Q., Zhang Y. (2014). Early and midterm outcomes of hemiarch replacement combined with stented elephant trunk in the management of acute DeBakey type I aortic dissection: Comparison with total arch replacement. J. Thorac. Cardiovasc. Surg..

[20] Roselli E.E., Idrees J.J., Bakaeen F.G., Tong M.Z., Soltesz E.G., Mick S., Johnston D.R., Eagleton M.J., Menon V., Svensson L.G. (2018). Evolution of simplified frozen elephant trunk repair for acute DeBakey type I dissection: Midterm outcomes. Ann. Thorac. Surg..

[21] Chen Q., Cheng F., Chen T., Zhao F., Jiang N. (2016). Ascending aorta replacement combined with open placement of triple-branched stent graft and total arch replacement combined with stented elephant trunk implantation for treating type A aortic dissection. Int. J. Clin. Exp. Med..

[22] Bertoglio L., Katsarou M., Loschi D., Rinaldi E., Mascia D., Kahlberg A., Lembo R., Melissano G., Chiesa R. (2020). Elective multistaged endovascular repair of thoraco-abdominal aneurysms with fenestrated and branched endografts to mitigate spinal cord ischaemia. Eur. J. Vasc. Endovasc. Surg..

[23] Kreibich M., Berger T., Rylski B., Chen Z., Beyersdorf F., Siepe M., Czerny M. (2020). Aortic reinterventions after the frozen elephant trunk procedure. J. Thorac. Cardiovasc. Surg..

[24] Di Bartolomeo R., Pantaleo A., Berretta P., Murana G., Castrovinci S., Cefarelli M., Folesani G., Di Eusanio M. (2015). Frozen elephant trunk surgery in acute aortic dissection. J. Thorac. Cardiovasc. Surg..

[25] Grabenwoger M., Mach M., Machler H., Arnold Z., Pisarik H., Folkmann S., Harrer M.-L., Geisler D., Moidl R., Winkler B. (2021). Taking the frozen elephant trunk technique to the next level by a stented side branch for a left subclavian artery connection: A feasibility study. Eur. J. Cardiothorac. Surg..

[26] Ince H., Rehders T.C., Petzsch M., Kische S., Nienaber C.A. (2005). Stent-grafts in patients with marfan syndrome. J. Endovasc. Ther..

[27] Ma W.G., Zhang W., Zhu J.M., Ziganshin B.A., Zhi A.H., Zheng J., Liu Y.M., Elefteriades J.A., Sun L.-Z. (2017). Long-term outcomes of frozen elephant trunk for type A aortic dissection in patients with Marfan syndrome. J. Thorac. Cardiovasc. Surg..

[28] Pellenc Q., Girault A., Roussel A., De Blic R., Cerceau P., Raffoul R., Milleron O., Jondeau G., Castier Y. (2020). Optimising aortic endovascular repair in patients with Marfan syndrome. Eur. J. Vasc. Endovasc. Surg..

[29] Shrestha M., Martens A., Kaufeld T., Beckmann E., Bertele S., Krueger H., Neuser J., Fleissner F., Ius F., Alhadi F.A. (2017). Single-centre experience with the frozen elephant trunk technique in 251 patients over 15 years. Eur. J. Cardiothorac. Surg..

[30] Etz C.D., Zoli S., Mueller C.S., Bodian C.A., Di Luozzo G., Lazala R., Plestis K.A., Griepp R.B. (2010). Staged repair significantly reduces paraplegia rate after extensive thoracoabdominal aortic aneurysm repair. J. Thorac. Cardiovasc. Surg..

[31] Kremer J., Preisner F., Dib B., Tochtermann U., Ruhparwar A., Karck M., Farag M. (2019). Aortic arch replacement with frozen elephant trunk technique—A single-center study. J. Cardiothorac. Surg..

